# Optimization of nutritional and functional qualities of local complementary foods of southern Ethiopia using a customized mixture design

**DOI:** 10.1002/fsn3.2663

**Published:** 2021-11-22

**Authors:** Dagem Alemayehu Ayele, Tadesse Fikre Teferra, Jan Frank, Samson Gebremedhin

**Affiliations:** ^1^ School of Nutrition, Food Science and Technology Hawassa University Hawassa Ethiopia; ^2^ Department of Food Biofunctionality Institute of Nutritional Sciences University of Hohenheim Hohenheim Germany; ^3^ School of Public Health Addis Ababa University Addis Ababa Ethiopia

**Keywords:** child nutrition, fortified food, infant, minerals, product optimization, vitamins

## Abstract

Commercially produced complementary foods are inaccessible to rural households in Ethiopia. This study aimed to optimize the nutritional and functional properties of local complementary foods using flours of the following locally available crops: maize, red kidney bean, *kocho*, and pumpkin fruit. Ten formulations were generated using a customized mixture design. A five‐point hedonic scale was used for the determination of organoleptic properties, and standard methods were used for the analyses of nutritional composition and functional properties. The flours were mixed in the range of 20%–30% for *kocho*, 10%–25% for pumpkin fruit, 10%–40% for red kidney bean, and 15%–30% for maize. Optimal nutritional and functional properties were obtained using 33.5% *kocho*, 22.5% maize, 17.5% pumpkin, and 26.5% red kidney bean. Optimal values for functional properties were 0.86 g/ml, 5.94 ml/g, 4.14 ml/g, 2.96 g/g, 5.0 ml/g, and 1225.3 cP for bulk density, water absorption capacity, oil absorption capacity, swelling capacity, swelling index, and viscosity, respectively. All formulations were within acceptable limits with scores ranging from 3.00 to 4.32 on a scale of 5. The inclusion of 25% pumpkin fruit flour and other ingredients between 20% and 30% increased the pro‐vitamin A carotenoid and vitamin E contents of the composite flours. Aside from optimization, a higher concentration of limiting amino acids was achieved with 40% kidney beans and 15%–25% of the other ingredients. The mineral contents improved with increasing pumpkin, kidney bean, and *kocho*. To sum up, the nutrient quality, energy density, and functional quality of complementary foods can be optimized at a low cost using local ingredients.

## Graphical abstract

This research aimed to formulate acceptable, nutrient and energy‐dense complementary foods using locally available crops with an optimized custom mixture design so as to combat protein‐energy malnutrition and enhance the nutrient intake of infants.

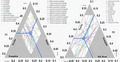

## 1 BACKGROUND

Inadequate feeding practices are a major contributor to morbidity, mortality, as well as growth and development impairment in infants and young children (Abeshu et al., 2016; Millward, 2017). Globally, undernutrition contributes to nearly 50% of deaths among children under 5 years of age (UNICEF/ChildInfo, 2018). Although Ethiopia has made some progress toward achieving the target for reducing wasting and stunting, 7% and 37%, respectively, in children younger than 5 years, the value is higher than the average (6.4%) for the African region (DHS & ICF, 2021; Global Nutrition Report, 2020).

Complementary feeding can play an important role in bridging the nutrient and energy gaps during the transition phase from exclusive breastfeeding to family foods. Because breast milk alone falls short of meeting all the nutrient requirements for optimal growth of infants, the WHO and UNICEF recommend that children should be introduced to nutritious and safe complementary foods at 6 months of age alongside continued breastfeeding (Beyene et al., 2015; Prell & Koletzko, 2016). In low‐income countries, inadequate nutrient intake from suboptimal complementary feeding in combination with a high incidence of infections at an early age is the major cause of high stunting rates and impaired development (Masuke et al., 2021). Most rural households in these countries do not have access to animal protein or fortified foods (Hailegebriel, 2020). Consequently, many resource‐poor families rely on plant‐based diets for their infants, which are usually based on a single type of cereal grain but prepared through different methods of cooking.

Achieving dietary diversity is a major challenge among resource‐poor settings in the developing world (Bedada et al. 2020; Bosha et al. 2019), particularly for children in rural Ethiopian households (Girma et al., 2015). In Ethiopia, only 7% of children aged 6–23 months satisfied the minimum standards for all the three IYCF practices and only 14% of children had an adequately diverse diet whereas 45% had been fed the minimum number of times appropriate for their age (CSA and ICF, 2017).

Starchy staples, including cereals and foods from *Ensete ventricosum* (*kocho*, *bulla*, and *amicho*), are commonly used for infant food preparation in central and southern parts of Ethiopia, even though they are low in protein and micronutrients and therefore a probable contributor to chronic undernutrition (Frühauf, 2018). Pulses are another important group of crops growing in all parts of Ethiopia (Getachew, 2019) and may complement starchy staples in terms of their limiting amino acids. As cereals are high and legumes are low in sulfur‐containing amino acids and cereals are low and legumes are high in lysine, the combined protein from both sources is of high biological value (Mariola Staniak and Bojarszczuk, 2014). However, due to a lack of awareness in the population, pulses are not commonly used in the preparation of complementary foods. Also, locally available pigmented vegetables, such as pumpkin (*Cucurbita pepo* L.), which contains a high amount of provitamin A carotenoids and tocopherols (vitamin E) (Kulczynski and Gramza‐Michałowska, 2019; Mahmoud et al. 2017), have been overlooked for their use in complementary foods.

We, therefore, hypothesized that it is possible to develop acceptable complementary food from locally available plant food sources to fill the nutrient and energy gaps in infants and young children in southern Ethiopia by making use of a customized mixture design.

## 2 MATERIALS AND METHODS

### 2.1 Experimental materials

Experimental materials for complementary food formulation were selected based on local availability, cultural acceptability, nutritional composition, and affordability. Red kidney bean (*Hawassa Dume Variety*), white maize (*DH‐540 Variety*), pumpkin (*Cucurbita pepo L*.), and a traditional processed product called *kocho* made from the corm of false banana (*E*. *ventricosum*) were all obtained from the local market of *Boricha* district located in *Sidama* Regional State, southern Ethiopia.

### 2.2 Material preparation

#### 2.2.1 Red kidney bean flour

Kidney beans were germinated to increase the bioavailability of minerals, the concentration of vitamin B (Oldewage‐Theron et al., 2015), nutrient density, and reduce the bulkiness of the complementary food (Nkhata et al., 2018). Bean flour was prepared as described by Kassegn et al. (2018).

In brief, the kidney beans were cleaned and soaked in tap water (1:5, w/v) at 24°C for 12 hr. The water was drained and the beans were kept moist by covering with a moist muslin cloth in the dark at an average temperature of 22.5°C for 48 hr. The germinated beans were sun‐dried for two consecutive days at an average temperature of 23°C, after which the sprouts were removed by abrasion. The dried beans were cooled, de‐hulled using a mini grinding miller (Model: Alvan Blanch, Mini 9E), and subsequently milled to flour using a high‐speed multifunction comminutor (Model: Ririhong, China), passed through a 710‐μm sieve and packed using polyethylene bags, and then stored under cold and dry conditions until required for formulation.

#### 2.2.2 Maize flour preparation

Using the same method discussed under section 2.2.1, maize grains (*Zea mays* L.) were germinated. The grains were first winnowed and then hand‐sorted to remove stones, leaves, and stalks, broken, undersized, and immature grains. The flour was prepared as described by Onwurafor et al. (2017). Briefly, the cleaned maize was steeped in cold tap water (1:3 w/v) for 3 hr. The steeped grains were then spread in a dark room for germination for 2 days and sun‐dried for another 2 days. The dried grains were gently pounded using a wooden mortar and pestle to remove a substantial amount of the pericarp as well as shoots and rootlets. The dry maize was then milled into flour and packed in polyethylene bags and stored at a temperature of 2**°**C until required for formulation.

#### 2.2.3 *Kocho* flour preparation

*Kocho* is an anaerobically fermented traditional food product extracted from the corms of the *E*. *ventricosum* plant (Gebremeskel et al., 2018). Its flour was prepared as described previously by Getaneh et al. (2017). In brief, traditionally prepared *kocho* mass was purchased from a local market and manually cut, mixed, squeezed, and sun‐dried for 22 hr at an average temperature of 24°C. The dried *kocho* flour was passed through a 710‐µm sieve to reduce fiber and stored at 2°C in polyethylene bags until required for product formulation.

#### 2.2.4 Pumpkin flour preparation

Pumpkin pulp (*Cucurbita pepo* L.) flour was prepared as described by Usha et al. (2010). In brief, the pumpkin fruit was washed under cold running tap water, peeled, halved, and seeds were completely removed. The flesh of the fruit was cut into even slices of 2‐mm thickness and finely chopped using an electric mini chopper (Model: Philips, HR776, China). The pieces were then dried at 60°C for 1 hr and milled using a fluid‐bed dryer (Sherwood Scientific, 230VAC, 50HZ); the powder was packed in polyethylene bags and stored in a dark and cold room until required for formulation.

### 2.3 Experimental design

A 10‐point customized mixture design (Table 1) with three center points was developed with JMP statistical software (a SAS Co. Product) to optimize nutrient composition (proximate, minerals, vitamins A and E) and functional properties (oil absorption capacity, water absorption capacity, swelling capacity, swelling index, and bulk density) of complementary foods. The optimal ranges of the combinations of flours of these crops were analyzed and reported as mixture profilers from all possible combinations.

**TABLE 1:** A 10‐level mixture experimental design with three center points. Values represent percentages based on the weight of the flours

Formula code	Kocho%	Pumpkin %	Maize %	Kidney bean %
R1	35	17.5	22.5	25
R2	35	10	15	40
R3	20	25	30	25
R4	20	25	15	40
R5	50	10	30	10
R6	35	17.5	22.5	25
R7	35	17.5	22.5	25
R8	20	10	30	40
R9	35	17.5	22.5	25
R10	50	25	15	10

### 2.4 Preparation of composite complementary flour

The dried flours were mixed at different proportions to make composite complementary flours based on a 10‐point customized mixture design (Table 1). The flour was mixed with a manual flour mixer for 5 min and porridges from composite flours were prepared as described (Mezgebo et al., 2018). For optimization purposes, customized mixture experimental design requires setting lower, center, and upper limits. Subsequently, these values were set as indicated in Table 2 based on reference values from previous studies (Abebe et al., 2007; Bresciani & Marti, 2019; Kuliakina et al., 2020).

**TABLE 2:** Reference values used to set lower limit, center points, and upper limits of proportions for ingredients in the customized mixture experimental design

Ingredients	Lower limit		Center points		Upper limit		References
	Coded*	Actual^†^	Coded*	Actual	Coded	Actual	
*Kocho*	−1	0.20	0	0.35	+1	0.50	Abebe et al. (2007)
Pumpkin	−1	0.10	0	0.175	+1	0.25	Hels et al. (2004), Kuliakina et al. (2020)
Maize	−1	0.15	0	0.225	+1	0.30	Abebe et al. (2007)
Kidney beans	−1	0.10	0	0.25	+1	0.40	Bresciani and Marti (2019)

* Coded = for continuous variables; the Coding column property transforms data in the range researcher specify from –1 to +1 called (DOE coding).

^†^ Actual =proportion of ingredients in fractions based on dry weight of used to develop composite flour in previous reference studies.

### 2.5 Quantification of macro‐ and micronutrients

#### 2.5.1 Proximate composition

Proximate composition (crude protein, total ash, and crude fat) of the optimized flour was determined in duplicate following the methods of the Association of Official Analytical Chemists International (AOAC, 2000). The total carbohydrate (CHO) content was determined by the difference method (% CHO =100 − (% protein + % fat + % ash + % crude fiber + % moisture)) as indicated by Merrill and Watt (1973).

The moisture content of the sample was determined after drying using the hot air oven method. The gross energy content of composite flours was estimated based on guidelines from Codex Alimentarius (FAO/WHO, 2015). Calories were calculated from fat, carbohydrate, and protein contents using the Atwater's conversion factors (4 kcal/g for protein and carbohydrates; 9 kcal/g for fat; FAO, 2003).

#### 2.5.2 Dietary minerals

Iron (Fe), zinc (Zn), potassium (K), natrium (Na), phosphorus (P), calcium (Ca), and magnesium (Mg) contents in 250 mg of samples were determined by ICP‐OES after microwave‐heated nitric acid digestion using an ultraclave according to the “VDLUFA‐Methodenbuch Band II.1, 8.10, 2007: Bestimmung von Mikronährstoffen in Düngemittelextrakten, ICP‐OES Methode” as previously described by Stuetz et al. (2012) and Noelle et al. (2020).

The analyses of minerals were performed at the Core Facility Hohenheim (CFH) of the University of Hohenheim, Stuttgart, Germany. All parameters were determined in duplicate.

The analyses were performed using slightly modified versions described by VDLUFA (2011). Briefly, the samples were moistened with water and nitric acid (HNO_3_) was added. Digestion was carried out on a Microwave Digestion System. After digestion, the samples were filled up with water and then measured using ICP‐OES (inductively coupled plasma optical emission spectrometry) (Agilent 5110 ICP‐OES, Agilent, United States).

#### 2.5.3 Limiting amino acids

Samples were selected for amino acid profiling based on their mineral concentration. Iron and zinc concentrations were the main factors used to screen samples for amino acid analyses. Samples containing relatively higher concentrations of these minerals were selected, namely, R1, R3, R4, R10, and R14.

Amino acids in the composite flour samples were determined in duplicate according to the commission regulation (EC) No 152/2009, annex III F: CELEX No 02009R0152‐20,130,212 (https://eur‐lex.europa.eu/eli/reg/2009/152/2013‐02‐12). Samples (2 g) were defatted with 150 ml of petroleum ether (boiling range 40–60°C). An aliquot (~1 g) was oxidized with performic acid–phenol mixture (at 0°C in an ice bath and then 16 hr in a refrigerator) for the analysis of the sulfur‐containing cysteine and methionine, while tyrosine and other amino acids (except tryptophan) were analyzed in unoxidized samples. All samples were hydrolyzed with hydrochloric acid (6 M HCl containing 1 g phenol/L) at 110°C (mixture temperature) for 23 hr, separated by ion‐exchange chromatography (oxidized feedstuff column, type cation exchanger resin, 20 × 4.6 mm column), postcolumn‐derivatized with ninhydrin and photometrically detected (570 nm for primary amino acids and 440 nm for secondary amino acids) using an amino acid analyzer (Biochrom 30, Biochrom Ltd., Cambridge, England).

An aliquot (0.5–1.0 g flour) was hydrolyzed under alkaline conditions (using barium hydroxide solution and heated to 125°C for 16 hr) and analyzed for tryptophan by HPLC (Agilent Technologies 1200 Series). The internal standard α‐methyl‐tryptophan was added and tryptophan separated and determined by use of a Nucleodur C18 Pyramid column (EC 125/4, 5 µm, Macherey‐Nagel) and fluorescence detection (EX: 280 nm, EM: 356 nm).

#### 2.5.4 Carotenoids

Carotenoids were extracted and analyzed in triplicate by HPLC as previously described (Lux et al., 2020). Briefly, 100 mg of flour was weighed into a centrifuge tube. One milliliter of the internal standard β‐apo‐8'‐carotenal methyloxime (12 µl in100 mL ethanol), 1 ml butylated hydroxytoluol in ethanol (0.2 g/L), and 1 ml of 50% KOH solution were added, and samples were incubated for 30 min at 70°C in a shaking water bath. After saponification, samples were cooled on ice and 1 ml of glacial acetic acid and 2 ml of saline solution (15%, w/v) were added. Carotenoids were extracted twice with 1 ml of hexane/diethyl ether (50/50, v/v). The combined supernatants were evaporated and resuspended in 200 µl of eluent A/B. Separation and quantification were performed on a Shimadzu (Kyoto, Japan) Prominence HPLC equipped with an LC‐20 AT pump system (1.5 ml/min), a DGU‐14A degasser, a CTO‐10AS column oven (set to 40°C), and an SPD‐20 A UV/Vis detector (operated at 450 nm). Separation was achieved with gradient elution on a Develosil RP‐Aqueous C30 column (250 × 4.6 mm i.d., 5 μm particle size, Phenomenex, Aschaffenburg, Germany) equipped with a C30 guard column. Eluent A consisted of acetonitrile/methanol (70/30, v/v), and eluent B consisted of acetonitrile/1,4‐dioxane/methanol (37/60/3, v/v/v).

Gradient settings were as follows: 0%–15% B (5 min), 15%–100% B (20 min), isocratically held at 100% B (5 min), 100%–0% B (2 min) and held at starting conditions (3 min). Carotenoids were quantified against external authentic standards and corrected by internal standards.

#### 2.5.5 Vitamin E

Vitamin E congeners were quantified in triplicate as previously described (Grebenstein & Frank, 2012). In brief, about 200 mg flour was weighed into a glass tube and 2 ml of ethanol (containing 1% ascorbic acid (w/v)), 900 µl of H_2_O, and 600 µl of KOH were added and samples saponified for 30 min at 70°C. Samples were then extracted three times with 2 ml of hexane and in total 5 of 6 ml added hexane was collected and evaporated. Samples were dissolved in 100 µl of ethanol and analyzed on a Shimadzu (Kyoto, Japan) Prominence HPLC equipped with an LC‐20 AT pump, a DGU‐14A degasser, a CTO‐10AS column oven (set to 40°C), cooled autosampler SIL‐20 AC HT (4℃), and an RF‐20A fluorescence detector. Tocopherols and tocotrienols were separated on a Phenomenex KinetexTM PFP column (2.6 µm, 150 × 4.6 mm; Phenomenex) using methanol/H_2_O (85:15, v/v) as eluent at a flow rate of 1.2 ml/min. The fluorescence detector was operated at an excitation wavelength of 296 nm and an emission wavelength of 325 nm. Peaks were recorded and integrated using Lab solution LC software (Shimadzu, Kyoto, Japan) and quantified against external calibration curves.

### 2.6 Functional properties of developed composite flour

#### 2.6.1 Water and oil absorption capacity

Water absorption capacity was determined as previously described (Adegbanke et al., 2018). Briefly, samples (1 g) were weighed and mixed with 10 ml of distilled water for water absorption capacity or the same amount of sunflower oil for oil absorption capacity in a clean 50‐ml beaker and thoroughly stirred with a glass rod for 2 min. The suspension obtained was then centrifuged at 3555 rpm using a Table Top centrifuge (Model: PLC‐05 220V/50Hz, Taiwan) for 30 min, and the supernatant was measured in a 10‐ml graded cylinder. The density of water was taken as 1.0 g/cm^3^ and that of sunflower oil as 0.96 g/ml. Water/oil absorbed was calculated as the difference between the initial volume of water/oil added to the sample and that of the supernatant.

$$\text{WAC}\left(\text{mL}/g\right)=\frac{\text{volume of water used}‐\text{volume of water after centrifugation}\left(\text{mL}\right)}{\text{weight of sample}\left(g\right)}$$

$$\text{OAC}\left(\text{mL}/g\right)=\frac{\text{volume of oil used}‐\text{volume of oil after centrifugation}\left(\text{mL}\right)}{\text{weight of sample}\left(g\right)}$$

where WAC is the water absorption capacity and OAC is the oil absorption capacity.

#### 2.6.2 Bulk density

The bulk density of each sample was measured as previously described by Adegbanke et al. (2018). Briefly, a flour sample of 50 g was weighed into a 100‐ml glass measuring cylinder. The measuring cylinder was then tapped repeatedly on a firm pad on the laboratory bench until the constant volume was achieved, and the volume was recorded. Bulk density (g/ml) was then calculated as:

$$\text{BD}\left(g/\text{ml}\right)=\frac{\text{weight of sample}\left(g\right)}{\text{volume of sample}\left(\text{ml}\right)}$$

where BD is the bulk density.

#### 2.6.3 Swelling index

The swelling index is the amount of water‐soluble solids per unit weight of the sample. It was measured as previously described by Adegbanke et al. (2018). Briefly, 3 g sample was transferred to a 50‐ml graded cylinder, and 30 ml of distilled water was added. The cylinder was swirled and then allowed to stand for 60 min at room temperature. The volume change was registered and the swelling index was calculated as:

$$\text{SI}\left(\text{ml}/g\right)=\frac{\text{volume after soaking}‐\text{volume before soaking}\left(\text{ml}\right)}{\text{Original weight of sample}\left(g\right)}$$

where SI is the swelling index.

#### 2.6.4 Swelling capacity

The gel (substance left after discarding the supernatant) obtained from the swelling index analysis was used to calculate the swelling capacity of the flour samples. The computational formula is given as:

$$\text{SC}\left(g/g\right)=\frac{\text{Weight of gel}\left(g\right)}{\text{weight of sample}\left(g\right)}$$

where SC is the swelling capacity.

#### 2.6.5 Viscosity

Porridge samples were prepared from each formula by cooking a mixture of flour and water (1:3, by volume) for 10 min. The cooked porridge was transferred to a 250‐ml beaker and placed in a water bath (Model: Kottermann D 3165, Hänigsen, W. Germany) maintained at 40°C. A Brookfield viscometer (Model: RVDV‐IT) was used to measure the porridge viscosity (in centipoises, cP) using spindle number 6 at a shear rate of 50 rpm as described (Tizazu et al., 2010).

### 2.7 Calculation of nutrient and energy densities

The nutrient density of the optimized composite flour was calculated using the results from the laboratory analyses described above and divided by the respective calculated energy and given as g/100 kcal. Energy density was calculated by dividing the energy content of the formulated composite flour by 100 and given as kcal/g (WHO/UNICEF, 1998). Bioconversion of pro‐vitamin A carotenoids into retinol equivalent was calculated as recommended by FAO & WHO (1998).

### 2.8 Sensory evaluation

Sensory evaluation for acceptability of the optimized porridge sample was conducted at *Boricha* district *Sidama* regional state, southern Ethiopia. Infants and young children are not matured enough to involve in sensory and consumer research on account of inability to communicate verbally, limited cognitive abilities, low sensitivity, and very low attention period (Lawless and Lawless, 1998).

Thirty‐two healthy and untrained panelists (mothers/caretakers and child pairs) were screened for the sensory evaluation while applying the lottery method. Following orientation, coded and freshly cooked samples were served in a white plastic cup along with bottled water to cleanse their palate between samples and during evaluation of the sensory attributes of porridge. The products were assessed for their appearance, texture, aroma, taste, and overall acceptability based on five‐point hedonic scales, where 1 = Dislike extremely, 2 = Dislike moderately, 3 = Neither like nor dislike, 4 = Like moderately, and 5 = Like extremely.

### 2.9 Statistical analyses

Statistical analyses were performed using JMP Pro, version 13, statistical software package (SAS Co., Cary, NC, USA). Lack of fit and model significance were used for checking the appropriateness of the Mixture Design for the proximate and functional properties of the optimized local complementary foods. Mean values for replicated measurements were computed and compared using one‐way ANOVA and statistically significant differences (*p* <.05) for the parameters other than the proximate and functional properties. Normality and uniformity of variance (Brown–Forsythe test) were assessed to check the fitness of the data for ANOVA without needing transformation. Differences between mean values were computed using Tukey honestly significant difference (HSD) test. Results were expressed as least square means ± standard error.

## 3 RESULTS

### 3.1 Dietary minerals

**FIGURE 1:**
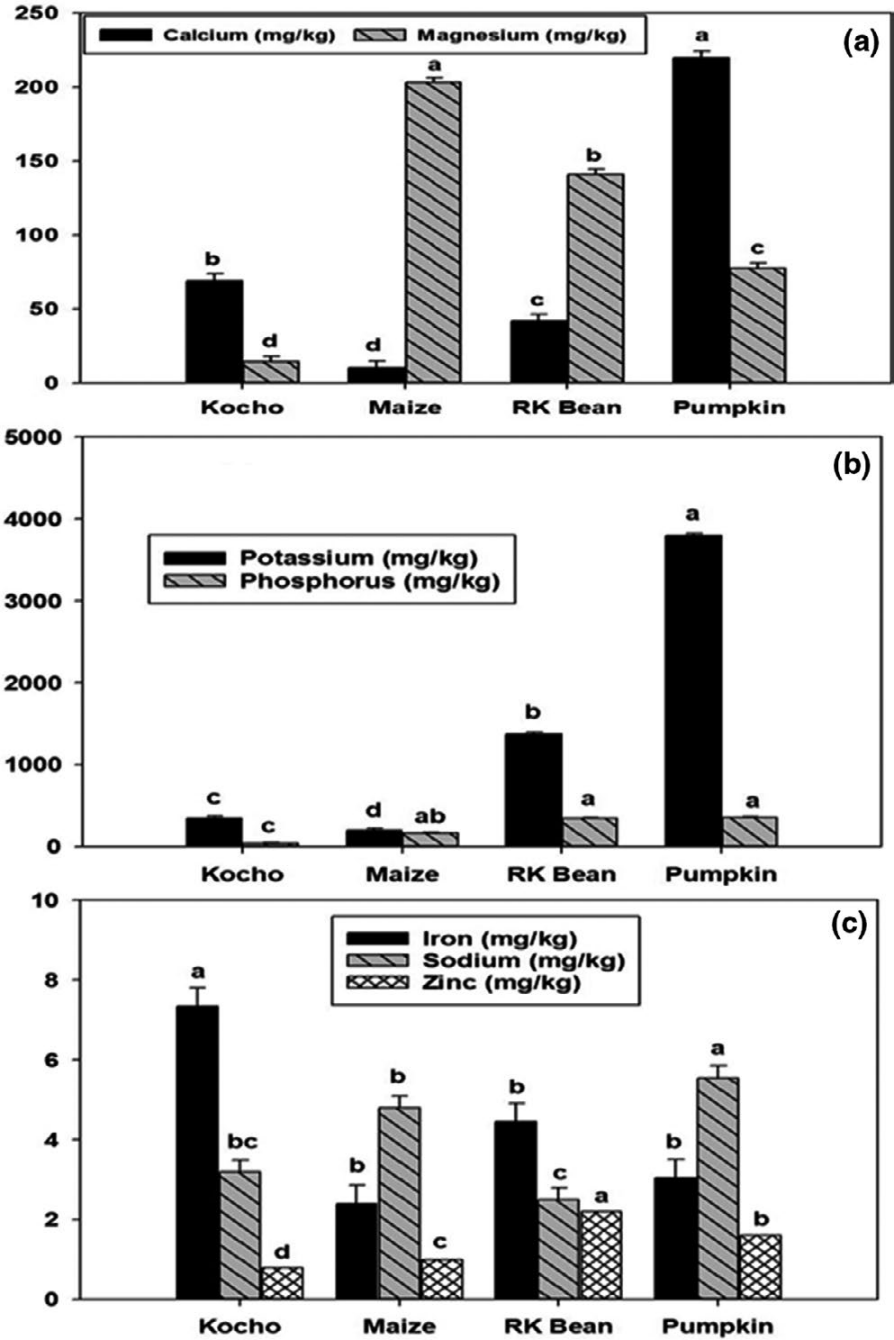
Concentrations of selected minerals in the *kocho*, maize, red kidney beans (RK bean), and pumpkin flours: [a] calcium and magnesium; [b] potassium and phosphorus; and: [c] iron, sodium, and zinc. Values are arithmetic means; error bars represent standard errors. For each mineral, bars not sharing common superscript letters are significantly different (*p* <.05)

The highest concentrations of calcium, phosphorus, and potassium were present in the pumpkin flour (Figure 1). The second‐highest potassium and zinc concentrations were measured in the kidney bean flour. *Kocho* had the highest content of iron of all flours. The inclusion of pumpkin and red kidney beans in the local complementary foods increased the mineral and micronutrient contents better than the other ingredients. The individual mineral contents are presented in the mixture profiler (Figure 4).

The calcium content of the composite flour significantly increased with the amount of *kocho* and pumpkin. Iron also increased with the addition of *kocho* and decreased with maize. Phosphorus decreased with the *kocho* and slightly increased with the amount of kidney beans in the composite flours. Zinc also decreased with increasing amounts of *kocho* and slightly increased with the other ingredients in the formulation (Figure 4; Table 3).

**TABLE 3:** Mean concentrations, standard error of the mean, and standard deviations of values measured for minerals (mg/100 g dry weight) in composite flours and major ingredients as determined by ICP‐MS. Values not sharing a common superscript letter are significantly different (*p* < .05)

Minerals	Formula code														SD
	R1	R2	R3	R4	R5	R6	R7	R8	R9	R10	R11	R12	R13	R14	
Ca	77.3 cd	63.6^d^	78.6 cd	79.1 cd	65.2^d^	73.4^d^	91.7^c^	64.0^d^	78.3 cd	119.4^b^	69^d^	10.3^f^	41.9^e^	220^a^	46.8
Fe	5.0^bc^	4.9^bc^	4.1^bcd^	4.4^bcd^	4.9^bc^	4.6^bcd^	4.4^bcd^	3.5^cde^	3.5^cde^	5.2^b^	7.4^a^	2.4^e^	4.5^bcd^	3.1^de^	1.2
K	1700^bcd^	1342.5^def^	1996^bc^	2109^b^	1108^ef^	1554^cde^	1264^def^	1061^f^	1139^ef^	1444^def^	352^g^	203^g^	1377^def^	3802^a^	855
Mg	66.9^bcd^	73.2^bc^	59.9^d^	77.1^b^	35.2^e^	58.8^d^	67.0^bcd^	77.9^b^	63.6 cd	58.3^d^	14.3^f^	68^bcd^	141.0^a^	77.2^b^	27.5
Na	4.4^abc^	3.1 cd	4^bcd^	3.5^bcd^	3.3 cd	3.4^bcd^	4.0^bc^	3.2 cd	3.9^bcd^	4.5^abc^	3.2 cd	4.8^ab^	2.5^d^	5.6^a^	0.8
P	226.9^bc^	218.0 cd	226.6^bc^	260.3^b^	129.6^f^	194.0^cde^	187.1^de^	205^cde^	179.1^e^	172.6^e^	41.8^g^	169^e^	348^a^	359^a^	80.1
Zn	1.5^a^	1.5^a^	2.4^a^	1.6^a^	1.0^a^	1.3^a^	1.2^a^	1.3^a^	1.1^a^	1.2^a^	0.8^a^	1.0^a^	2.2^a^	1.6^a^	0

Note

Formula code (R1–R10) is described in detail under Table 1 above. R11 = (100% K), R12 = (100% M), R13 = (100%RKB), R14 = (100% P).

### 3.2 Nutrient and energy density of composite flours

The nutrient and energy density of optimized flours are indicated in Table 4. High calcium density (27.7 mg/100 kcal) was observed in the sample in which the proportion of *kocho* and maize flours were high 35% and 22.5%, respectively, while the lowest value (19.3 mg/100 kcal) corresponded to the samples with a lower proportion of maize 15% in the mixture and the optimal value was 24.1%. The optimal value for dietary iron was 1.3 mg/100 kcal, and the high density of 1.5 mg/100 kcal corresponded to the formulation containing the highest level of *kocho* (35%). On the contrary, the lowest value of iron density (1.1 mg/100 kcal) was observed for the samples in which the proportion of *kocho* was low (20%).

**TABLE 4:** Optimal, highest and lowest energy, and nutrient density in dry weight of optimized mix flour after result from laboratory analyses analyzed by JMP profiling tools

Variable	Low value	High value	Optimal value
Ca (mg/100 kcal)	19.3	27.7	24.1
Fe (mg/100 kcal)	1.1	1.5	1.3
K (mg/100 kcal)	321.5	639	450.4
Mg (mg/100 kcal)	18.2	35.2	19.6
Na (mg/100 kcal)	0.9	1.3	1.2
P (mg/100 kcal)	56.2	79.7	58.0
Zn (mg/100 kcal)	0.3	0.7	0.4
Vitamin A (µg RE/100 kcal)	34.9	79.6	63.1
Vitamin E (mg/100 g)	10.7	51.0	27.1
Protein (g/100 kcal)	1.7	3.6	3.0
Energy (kcal/g)	3.3	3.3	3.3

Note

Mixture composition of each formula code (R1–R10) is described in detail under Table 1 above.

The highest zinc density (0.7 mg/100 kcal) was recorded in the sample mixture containing a high proportion of pumpkin, maize 20%, and 30%, respectively, correspondingly lowest value of 0.3 mg/100 kcal was observed in the samples with the lowest 10% pumpkin mix optimized formulation and optimal value was 0.4 mg/100. Lowest vitamin A and E density 35.5 µg RE/100 kcal and 10.7 mg/100 kcal respectively associated with a high proportion of *kocho*, kidney bean 35% and 40% respectively (data not shown), correspondingly their high density 79.6 and 51.0 mg/100 kcal respectively corresponds to samples contained a combination of maize and pumpkin in the lowest proportion 15% and 10% respectively (data not shown), and optimal values were 63.1 µg RE/100 and 27.1 mg/100 kcal respectively. Magnesium, potassium, and phosphorous densities observed high 23.4, 639, and 79.7 mg/100 kcal respectively in the same samples containing the highest proportion of pumpkin and red kidney bean 25% and 40% respectively. A high protein density of 3.6 mg/100 kcal was observed in the samples containing the highest proportion of kidney bean (40%). Finally, the energy density was almost similar to 3.3 kcal/g in all formulations.

### 3.3 Limiting amino acid contents in selected composite flours

**FIGURE 2:**
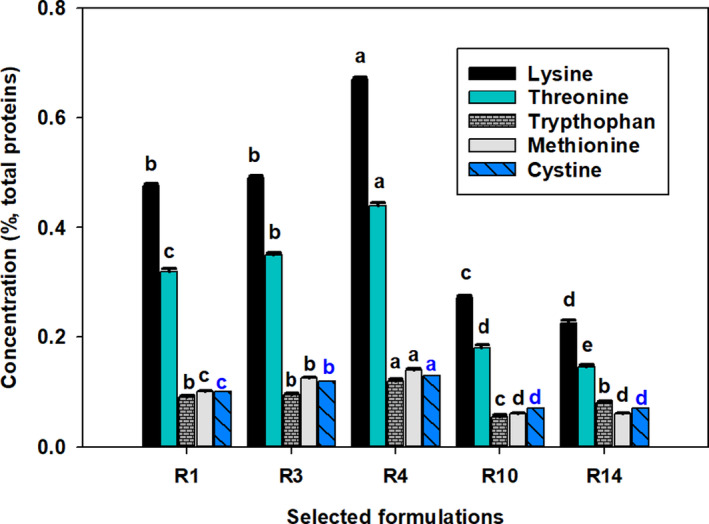
Concentrations (least square mean, error bars show *SEM*) of limiting amino acids in selected composite flours. For each amino acid, bars not sharing a common letter are significantly different (*p* <.05). Flour compositions for sample coding R1–R10 are given under Table 1; R14 = (100% pumpkin fruit flour)

The concentration of lysine, cysteine, tryptophan, methionine, and threonine in the current optimized composite flours was observed to be as high as 0.67%, 0.13%, 0.12%, 0.14%, and 0.44%, respectively, and this was observed in the sample containing the highest proportion of red kidney bean flour (40%) and lowest value of maize flour (15%). On the other hand, low values of lysine, cysteine, tryptophan, methionine, and threonine were recorded in the sample containing a low proportion of red kidney bean (25%) and a high proportion of maize flour (22.5%). Likewise, the lowest values of the limiting amino acids were observed when 50% of *kocho* and 50% of pumpkin flours were mixed in the optimized formulas (Figure 2).

### 3.4 Functional properties of optimized composite flours

**FIGURE 3:**
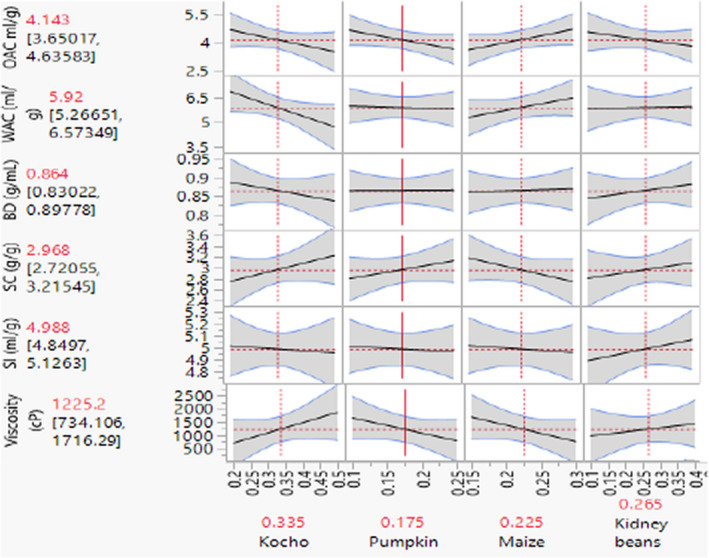
Prediction profiler of functional properties for optimizing flour mix. Concentration of major ingredient weight in fractions (horizontal line); maximum and minimum values for functional properties of optimize mix flour in brackets (vertical lines); and optimal values for each ingredients and functional properties are written in red color

**FIGURE 4:**
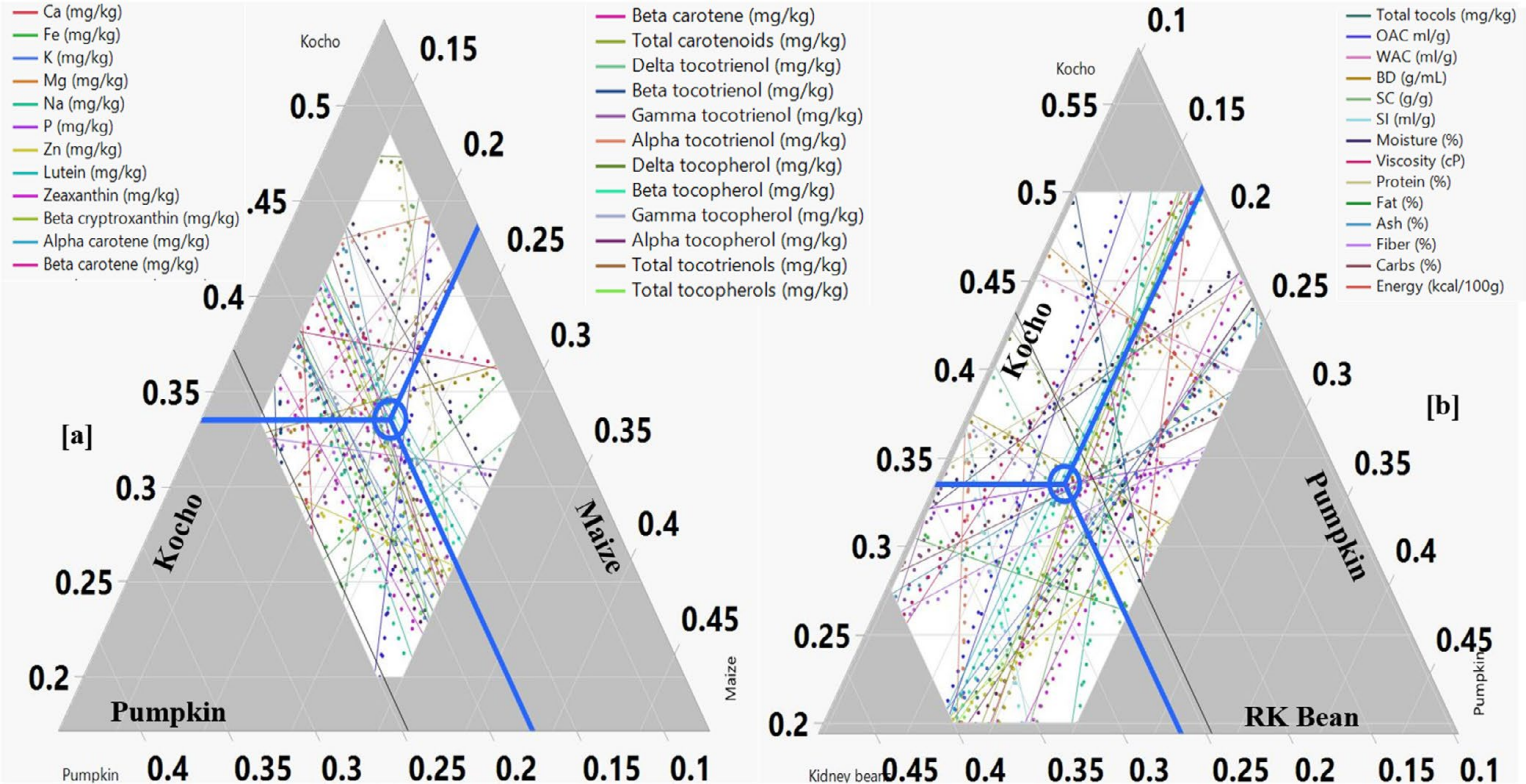
Optimal formulation (blue circle) for proximate composition, functional properties, and micronutrient contents of complementary foods in fractions based on their dry weight. [a] Combinations of *kocho*, pumpkin, and maize; [b] combinations of pumpkin, *kocho*, and RKB. The bigger triangular region is the design space, whereas the white region satisfies all the constraints in the DOE and the lines in the white region indicate the trend of increasing or decreasing nutritional and functional properties; blue circles indicate the optimal ratio for each ingredient. RKB = red kidney bean; and DOE = design of experiment

The functional properties (water absorption capacity, oil absorption capacity, swelling capacity, swelling index, and viscosity) of the developed composite flours are presented in the mixture profiler (Figure 4) and in the prediction profiler (Figure 3). Both water absorption capacity and bulk density were slightly decreased with increasing amounts of *kocho*, and significantly increased with red kidney bean proportion in the mixture (Figure 3).

Oil absorption capacity decreased with increasing contents of *kocho*, pumpkin, and kidney bean, but slightly increased with that of maize. The swelling capacity increased with the amount of *kocho*, pumpkin, and kidney beans, but reduced with the proportion of maize in the flours. The swelling index increased with the portion of kidney beans but was not significantly affected by the other flours. Viscosity increased with increasing *kocho*, and decreased with increasing pumpkin and maize flour (Figure 3).

### 3.5 Sensory acceptability of the complementary foods

The mean sensory scores for cooked porridges using the developed local composite flours R1‐R10 ranged from 3.0 to 4.3 (average values) on a scale of 5 (Table 5). All porridges were accepted in terms of all the sensory attributes considered with the scores of 4 and 5 most frequently granted for overall acceptability. The scores for taste, texture, and overall acceptance seemed to increase together with the proportion of pumpkin flour, although there is no clear statistical segregation (*p* > .05).

**TABLE 5:** Sensory evaluation scores of porridges made from optimized *kocho*–maize–red kidney bean–pumpkin flours. Mean values not sharing a common superscript letter differ significantly (*p* < .05, one‐way analysis of variance)

Sensory attributes	Formula code								
	R1	R2	R3	R4	R5	R7	R8	R10	SE
Appearance	3.7^ab^	3.5^ab^	4.2^a^	3.9^a^	4.0^a^	3.9^a^	3.8^ab^	3.0^b^	0.2
Taste	3.6^a^	3.3^a^	4.1^a^	3.7^a^	3.5^a^	3.8^a^	3.4^a^	3.8^a^	0.19
Aroma	3.8^ab^	3.7^ab^	4.1^ab^	3.9^ab^	3.5^ab^	4.2^a^	3.4^b^	3.8^ab^	0.18
Texture	4.5^a^	3.9^ab^	4.3^a^	4.2^ab^	3.6^b^	4.0^ab^	3.9^ab^	3.7^ab^	0.16
Overall acceptability	3.9^a^	3.9^a^	4.2^a^	3.8^a^	3.7^a^	4.1^a^	3.7^a^	3.7^a^	0.0

Note

Compositions of formulations, coding (R1–R10), and abbreviation are explained in the footnote of Table 1. Thirty‐two untrained panelists were involved in the current sensory evaluation.

### 3.6 Optimal combination of formulated mix flour

The optimal proximate composition, functional properties, and micronutrient concentrations of the developed complementary flours were achieved with 33.5% *kocho*, 22.5% maize, 17.5% pumpkin, and 26.5% red kidney beans. The optimal composition is indicated by a circle around the juncture of three lines meeting from the three ingredients (Figure 4). The optimal proximate (crude protein, crude fat, total carbohydrate, and crude fiber) composition of the developed composite flour was determined and is shown in the mixture profilers (Figure 4).

The moisture content of the developed composite flour slightly increased with increasing amounts of starchy ingredients *(kocho* and maize) and decreased with the increasing content of kidney beans and pumpkin. The crude protein and ash contents slightly increased with the amount of pumpkin and kidney beans, and slightly decreased with increasing amounts of *kocho* and maize. Total carbohydrates increased with the content of *kocho* (Figure 4). Fats are not used by the local community for complementary foods and fiber is not recommended for child feeding as it increases the bulk of the food at the expense of important nutrients such as calories and micronutrients.

## 4 DISCUSSION

### 4.1 Contribution of optimized local complementary foods to the energy and macronutrient requirements of children

The highest energy density value for the formulated composite flour made from *kocho*, pumpkin, maize, and red kidney bean was 3.3 kcal/g (Table 4). According to the Codex standard for processed cereal‐based foods for infants and young children, the energy density of a cereal‐based complementary food should be ≥0.8 kcal/g (Commit Codex Alimentarius, 2006). Hence, our optimized composite flours can help to meet the minimum daily energy requirements for infants and young children aged 6–23 months but lower from another study for sweet potato‐based infant wean‐mix (4.4 kcal/g; Amagloh et al., 2011). The range of protein in the optimized formulations was 1.7–3.6 and the optimal value was 3.0 g/100 kcal (Table 4).

The Codex standard sets the upper threshold of protein requirements for infants and children at 5.5 g/100 kcal (Commit Codex Alimentarius, 2006); thus, our developed complementary food can also help to meet this requirement if 250‐ml cup of this meal is fed two to three times per day for children aged 6–8 months and three to four times per day for children aged 9–11 and 12–24 months accompanied by additional nutritional snacks and average breastfeeding (K. Dewey, 2000; WHO, 2009). These values were in agreement with teff‐soybean‐based complementary food by Tenagashaw et al. (2017). However, protein density and energy density in this study are slightly lower than those of local complementary foods from West Africa (protein 4.00 g/100 g; energy 4.00 kcal/g) reported by Onofiok and Nnanyelugo (1998) and Nigeria (protein 6.52 g/100 g; energy 4.0–4.2 kcal/g) as described by Oluwole Steve and Isaac Babatunde (2013). Alternatively, these values are higher than the energy and protein densities of local complementary foods from Ethiopia—0.48–0.53 kcal/g and 2.13–2.48 g/kcal, respectively (Abebe et al., 2006; Geleta et al., 2016).

Cereals and pulses differ in their limiting amino acids. The biological value of the protein can therefore be improved by combining both protein sources in a single meal (Hall et al., 2017; Sozer et al., 2017). This was considered in the development of the optimized formulations in this study.

### 4.2 Contribution of optimized local complementary foods to the micronutrient requirements of children

The vitamin A density of the optimized complementary food ranged from 34.9 to 79.6 and the optimal value was 63 μg RE/100 kcal; the current result was two‐fold higher when compared with the daily requirement of 31 μg RE/100 kcal for cereal‐based complementary foods for 6‐ to 23‐month‐old infants (K. G. Dewey & Brown, 2003). The current finding was higher than for complementary food made from orange‐fleshed sweet potato (24.55–42.81 RE/100 kcal; Tenagashaw et al., 2017). The extent of food processing and thermal treatment (cooking) in the preparation of plant‐based complementary food breaks the cell wall structure and releases carotenoids from complex food matrix. Both phenomena increase the bioaccessibility and bioavailability of carotenoids (Fernández‐García et al., 2012). In the same way, the presence of fat in a meal facilitates the incorporation of carotenoids into mixed micelles and therefore an intake of 3–5 g of fat is recommended to aid carotenoid absorption (Murkovic et al., 2002; Priyadarshani, 2017; Roodenburg et al., 2000). This can be achieved, for example, by feeding plant‐based complementary foods together with breast milk (Giugliani & Victora, 2000).

The calcium density in our optimized formulation was 24.1 mg/100 kcal (Table 4), which can meet 25% of the daily requirement as set by the WHO for infants aged 6–8 months and 32% for infants aged 9–11 months (K. G. Dewey & Brown, 2003) or nearly 50% of the minimum calcium intake for infants (50 mg/100 kcal; Koletzko et al., 2010).

The iron density in the optimized formulation was 1.3 mg/kcal, which does only meet 28.9% of the recommended iron density for complementary foods for 6‐ to 11‐month‐old infants of 4.5 mg/100 kcal (K. G. Dewey & Brown, 2003). The absorption of iron from plant‐based foods is low but can be enhanced by the simultaneous intake of vitamin C. Hence, the addition of locally available fruits, such as papaya and mango, and vitamin C‐rich vegetables, such as spinach, cabbage, and potato, is recommended for optimal iron absorption (Hurrell & Egli, 2010). The present result for iron and calcium density is in agreement with complementary foods in southern Ethiopia, but slightly lower than for zinc density (Geleta et al., 2016).

The zinc density in the optimized formulation was 0.4 mg/100 kcal (Table 4) and thus below the recommended value of 1.6 mg/100 kcal for 6‐ to 8‐month‐old infants. The Ca, Fe, and Zn contents of the individual flours used in the current formulation were in agreement with those reported in the literature, in particular for *kocho* (Abebe et al., 2007; Andeta et al., 2019; Atlabachew & Chandravanshi, 2008; Hailegebriel, 2020). To meet the daily requirements for Na, Mg, P, and K for infants and young children, it would be necessary to feed the optimized formulation two to three times per day.

### 4.3 Functional properties of the optimized flour mix

Functional properties are often overlooked in the development and optimization of complementary foods, with the major focus typically being on the nutrient composition. The functional properties of food matrices, however, are important determinants of nutrient and energy densities as well as stability and shelf life (Razjoo et al., 2021).

The water absorption capacity of 5.92 ml/g of the optimized formulated flour mixture (Figure 3) is lower than the legume‐based complementary food mentioned by Matewos (2017) but higher than millet–soy bean‐ as well as cassava‐rice‐based complementary foods formulated in Nigeria and Indonesia—2.19 and 2.63 g/g, respectively—by M. Zakari et al. (2018) and Julianti et al. (2014)), but in agreement with 5.46 ml/g pumpkin–sorghum‐based complementary food from Nigeria. The oil absorption capacity of the current optimized composite flour (4.14 ml/g; Figure 3) is much lower than quality maize‐based complementary food (8.85–10.8 ml/g; Berhanu et al., 2015).

Water absorption capacity depends on the ability of a polysaccharide or protein matrix to absorb, retain, and also physically entrap water against gravity and is strongly associated with flour thickness and viscosity (Traynham et al., 2007). A high water absorption capacity is an indicator of higher moisture in the matrix and the water will thus dilute the energy and nutrient content of the composite flour (Michaelsen et al., 2017).

Both the swelling capacity of 2.98 g/g and swelling index of 4.98 ml/g of the optimized flour (Figure 3) were higher than those of cereal‐nut‐based complementary foods—1.26 ml/g and 0.55 ml/g, respectively (Adegbanke et al., 2018). The optimal bulk density of the developed flour mix (0.86 g/ml; Figure 3) is in agreement with the bulk density of legume‐based and cereal‐legume‐based complementary foods (0.82 g/ml; Borbi et al., 2020; Ijarotimi & Keshinro, 2012), but lower than pumpkin‐based complementary food (0.46 g/ml; Bello et al., 2017). The variation in bulk density of foods could be due to the variation in starch and initial moisture content of the foods and increase correspondingly with the starch content (Iwe et al., 2016; Godswill et al., 2019). The high value of bulkiness is undesirable for complementary food due to the physiology of the alimentary canal and stomach capacity of the infant that is usually small to accommodate bulky food material (Adeyeye, 2009; Singh, 2013).

The optimal viscosity (1225.2 cP; Figure 3) of porridge made from the optimized flour mixture is lower than that of porridge from southern Ethiopia formulated from orange‐fleshed‐bean composite flour (2038 cP; Jemberu et al., 2016), but in agreement with sorghum‐based complementary food (1239.7 cP; Tizazu et al., 2010).

### 4.4 Sensory properties

The mean scores for sensory attributes for optimized porridge samples are indicated in Table 5. The appearance of porridge samples prepared from optimized composite flour ranged from 3.0 to 4.0, and a formulation containing the highest proportion of pumpkin and maize 25% and 30%, respectively, was liked most for its appearance. A similar result was reported by Olika et al. (2021) for optimized sorghum–soybean‐based flour gruel, which had exhibited no significant difference (*p* > .05) when *kocho* flour in the optimized mixture was increased from 20% to 50%. Differently, the mean taste score ranged from 3.3 to 4.1 and had no significant difference for all optimized formulations (*p* > .05).

The mean score for aroma ranged from 3.4 to 4.2 and showed no significant difference (*p* > .05) except in two formulas containing a high proportion of *kocho* (35%) and in a mixture containing a high proportion of kidney bean (40%) and a low proportion of pumpkin (10%). This is in agreement with Mekuria et al. (2021) for grain‐based weaning food in which aroma score ranged from 3.73 to 4.23. As regards the texture, the mean score for most of the formulations showed no significant difference (*p* > .05). The least preference showed on overall acceptability was observed in the porridge containing the highest proportion of kidney bean flour (40%); this could be due to the development of undesirable beany flavor masking the final product (Bresciani & Marti, 2019; Roland et al., 2017). The current optimized complementary food is also far better in all sensorial attributes than soya bean–moringa‐based complementary food formulated by Gebretsadikan et al. (2015).

In the most preferred formulation, each ingredient in the optimized mixture was proportionally equal; this probably made the product develop familiar sensory properties with the existing local complementary foods in the locality, though there was no significant difference (*p* < .05) among formulations for overall acceptability.

### 4.5 Limitations of the study

Due to budget limitations as well as COVID‐19–enabled travel restrictions, parameters such as food safety or microbial analyses and protein digestibility of the final products were not analyzed. For the same reasons, vitamins A and E as well as amino acids were not analyzed for all samples and their replications.

## 5 CONCLUSION

The presented data support that it is possible to develop acceptable, nutrient‐ and energy‐dense complementary foods using only locally available foods in southern Ethiopia, namely *kocho*, pumpkin, maize, and red kidney bean. We identified pumpkin fruit flour as an important source of provitamin A carotenoids and minerals that could help to combat vitamin A deficiency.

We further observed that by optimizing the ratios of kidney bean, maize, and *kocho* flours, the content of limiting amino acids can be increased and the protein and energy densities of complementary food are enhanced. To sum up, the use of locally available crops in the formulation of complementary food can be a successful strategy to combat protein energy and micronutrient malnutrition in infants and young children.

## AUTHOR CONTRIBUTION

**Dagem Alemayehu Ayele:** Conceptualization (lead); Data curation (equal); Formal analysis (supporting); Methodology (lead); Resources (lead); Writing‐original draft (lead); Writing‐review & editing (lead). **Samson Gebremedhin:** Conceptualization (equal); Data curation (supporting); Funding acquisition (lead); Methodology (supporting); Project administration (lead); Supervision (lead); Writing‐review & editing (supporting). **Jan Frank:** Conceptualization (equal); Data curation (supporting); Funding acquisition (lead); Methodology (equal); Project administration (lead); Supervision (lead); Writing‐review & editing (supporting). **Tadesse Fikre Teferra:** Conceptualization (equal); Data curation (equal); Formal analysis (lead); Methodology (equal); Software (lead); Supervision (supporting); Writing‐original draft (equal); Writing‐review & editing (supporting).

## ETHICAL APPROVAL

Ethical clearance for the sensory analyses was received from the Institutional Review Board of Hawassa University, College of Medicine and Health Science (Ref No. IRB/215/10). The authors declare no conflict of interest of any sort.

## INFORMED CONSENT

Informed verbal consent was obtained from each of the sensory panelists.

## Data Availability

The dataset supporting this study will be available from the corresponding author upon reasonable request.
